# The effect of the enhanced recovery after surgery program on radical cystectomy: a meta-analysis and systematic review

**DOI:** 10.3389/fsurg.2023.1101098

**Published:** 2023-05-19

**Authors:** Yongheng Zhou, Rongyang Li, Zhifeng Liu, Wenqiang Qi, Guangda Lv, Minglei Zhong, Xigao Liu, Meikai Zhu, Zhiwen Jiang, Shouzhen Chen, Benkang Shi, Yaofeng Zhu

**Affiliations:** ^1^ Department of Urology, Qilu Hospital of Shandong University, Jinan, China; ^2^ Department of Thoracic Surgery, Qilu Hospital of Shandong University, Jinan, China; ^3^ Department of Urology Surgery, Taian City Central Hospital, Taian, China

**Keywords:** enhanced recovery after surgery (ERAS), radical cystectomy, bladder cancer, systematic review, meta-analysis

## Abstract

**Background:**

Bladder cancer is the ninth most common malignant tumor worldwide. As an effective evidence-based multidisciplinary protocol, the enhanced recovery after surgery (ERAS) program is practiced in many surgical disciplines. However, the function of ERAS after radical cystectomy remains controversial. This systematic review and meta-analysis aims to research the impact of ERAS on radical cystectomy.

**Methods:**

A systematic literature search on PubMed, EMBASE, SCOPUS, and the Cochrane Library databases was conducted in April 2022 to identify the studies that performed the ERAS program in radical cystectomy. Studies were selected, data extraction was performed independently by two reviewers, and quality was assessed using a random effects model to calculate the overall effect size. The odds ratio and standardized mean difference (SMD) with a 95% confidence interval (CI) served as the summary statistics for the meta-analysis. A sensitivity analysis was subsequently performed.

**Results:**

A total of 25 studies with 4,083 patients were enrolled. The meta-analysis showed that the complications (OR = 0.76; 95% CI: 0.63–0.90), transfusion rate (OR = 0.59; 95% CI: 0.39–0.90), readmission rate (OR = 0.79; 95% CI: 0.64–0.96), length of stay (SMD = −0.79; 95% CI: −1.41 to −0.17), and time to first flatus (SMD = −1.16; 95% CI: −1.58 to −0.74) were significantly reduced in the ERAS group. However, no significance was found in 90-day mortality and urine leakage.

**Conclusion:**

The ERAS program for radical cystectomy can effectively decrease the risk of overall complications, postoperative ileus, readmission rate, transfusion rate, length of stay, and time to first flatus in patients who underwent radical cystectomy with relative safety.

**Systematic Review Registration:**

https://inplasy.com/, identifier INPLASY202250075.

## Introduction

Bladder cancer (BCa) is the ninth most common malignant tumor worldwide and the seventh cause of cancer death in men, causing more than 17,000 deaths in the United States in 2019 (1, 2). Radical cystectomy and lymphadenectomy are the gold standard for treating high-risk non–muscle-invasive and muscle-invasive BCa (3). Radical cystectomy is a complex procedure, usually accompanied by lymph node dissection and the choice of urinary diversion, resulting in many postoperative complications. With the advance in surgical modalities, such as robot-assisted radical cystectomy, intraoperative blood loss (IBS) and in-hospital stay have improved compared with traditional open radical cystectomy. However, the high-grade complication and mortality rates were similar between these two methods (4). For the complications after radical cystectomy, not only surgery but also preoperative and postoperative care was vital. Enhanced recovery after surgery (ERAS) is a tool to speed up patient discharge, restore body function smoothly, and reduce pain response, anxiety, and postoperative complications. Since its first application in colorectal surgery in the late 1990s (5), ERAS has been gradually developed and applied in other surgical specialties. An ERAS pathway optimizes preoperative, intraoperative, and postoperative elements, which include the improvement of oral mechanical bowel preparation, preoperative fasting, preoperative carbohydrate loading, analgesia, and mobilization, to speed up postoperative intestinal peristalsis and reduce postoperative complications (6).

To check the clinical value of ERAS, many scholars have done many clinical research and meta-analysis articles to investigate whether the variables, which include length of stay (LOS), postoperative complications rate, readmission rate, and mortality, would be improved after the implementation of ERAS. However, the results of these studies were inconsistent. A recent evidenced-based review and meta-analysis reported by Peerbocus and Wang (7) in 2021, which included 13 articles, one retrospective article, and one prospective article, demonstrated that the implementation of ERAS was beneficial for reducing LOS and the time to first defecation but was not well explained for readmission and overall complications due to limited data. To draw a convincing conclusion, we carried out a systematic review and meta-analysis to illustrate the impact of ERAS on radical cystectomy, especially on intraoperative and postoperative variables.

## Material and methods

This systematic review and meta-analysis was conducted in accordance with the Meta-analysis of Observational Studies in Epidemiology (MOOSE) guidelines (8) and the Preferred Reporting Items for Systematic Reviews and Meta-Analyses (PRISMA) statement (9) and registered as INPLASY202250075 at the International Prospective Register of Systematic Reviews (https://inplasy.com/).

### Databases and search strategy

This systematic review and meta-analysis is conducted using four online databases: PubMed, EMBASE, SCOPUS, and the Cochrane Library (from April 11, 2022, to April 13, 2022). The Medical Subject Headings (MESH) terms included in the search strategy were “urinary bladder neoplasms,” “radical cystectomy,” and “enhanced recovery after surgery,” and the free terms were searched in PubMed. Supplementary Table S1 shows the detailed search strategies for all databases. YhZ and RYL independently searched and cross-checked the article. Furthermore, the references of excluded articles were also independently researched to avoid the loss of important documents. Discrepancies between reviewers were resolved through discussion.

### Study selection and criteria

The inclusion criteria are as follows:

(I) P: patients with bladder cancer and undergoing radical cystectomy (laparoscopic radical cystectomy, open radical cystectomy, and robot-assisted radical cystectomy),

(II) I: involved patients who received an ERAS program [we recognized a total of 23 elements, of which 22 elements were confirmed from the guideline and one from a study of ERAS updates, encompassing all phases of perioperative care (pre-, intra-, and postoperative)],

(III) The ERAS program included at least eight elements that covered at least two phases of perioperative care,

(IV) C: include a traditional control group (non-ERAS) with at least three fewer elements than those of ERAS,

(V) O: reported at least one of the outcome measures mentioned above, and

(VI) Written in English

The exclusion criteria are as follows: (I) inappropriate article types, such as case reports, reviews, and conference abstracts; (II) no outcomes of interest present; and (III) not meeting the inclusion criteria and not being written in English.

### Endpoints and outcome measures

At least one of the following outcomes must be reported: LOS; time to first flatus, the passage of first stool, and time to normal diet and ambulation; intraoperative blood loss; operative time; readmission; postoperative ileus (POI); overall complication; 90-day mortality; urine leakage; and transfusion rate.

## Data extraction

YhZ and RYL independently reviewed and extracted data from the eligible studies to fill in the predefined form. The data to be extracted are as follows:

(I) publication data: authors, year, and country,

(II) baseline data: age, gender, study design, study period, ERAS elements, surgical approach, and the way of urethral diversion, and

(III) outcomes of interest: length of hospital stay; time to first flatus, the passage of first stool, normal diet, and ambulation; overall complication; transfusion rate; and mortality

Any disagreements were resolved through discussion.

### Quality assessment

The quality of included cohort studies was assessed using the Newcastle–Ottawa Quality Assessment Scale (NOS) (10), as shown in Tables 5A,B. We included studies with a scale score equal to or higher than 6 in our meta-analysis. In addition, the Cochrane risk-of-bias tool, which is in the Review Manager software (https://training.cochrane.org/online-learning/core-software/revman/revman-5-download), was used to evaluate the quality of randomized controlled trials (RCTs). YhZ and RYL independently assessed the quality of each study, and the disagreements concerning the quality assessment were resolved by a third investigator (WQ).

### Statistical analysis

The risk ratio (RR) with a 95% confidence interval (CI) was used to evaluate the effects of ERAS protocols on dichotomous data. The standardized mean difference (SMD) with 95% CI served as the appropriate statistic for continuous variables. If the median and range, rather than the mean and standard deviation (SD), were provided, the data were not transformed to mean and SD, as the guidelines of the Cochrane Collaboration showed that the extrapolation of SDs was only applicable to studies with large sample size and normal distribution of outcomes (10). The meta-analysis was not performed when the number of studies was very small (*n* < 5); instead, a qualitative summary was conducted.

The Cochrane *Q* test and *I*^2^ statistics were used to assess the heterogeneity level. An *I*^2^ of 25%, 50%, and 75% represented low, moderate, and considerable variance, respectively (11). The statistical significance was defined as a two-sided *P-*value < 0.05. We used the random effects models to estimate pooled effect sizes in order to reduce possible bias. Egger's test detected potential publication bias (12, 13). A significant publication bias was reported if Egger's *P-*value was <0.05.

A sensitivity analysis was performed to test the stability of pooled estimates through the deletion of individual studies sequentially. Our meta-analysis was confirmed to exhibit strong robustness if there was no material change between the adjusted and primary results (14).

All statistical analyses were conducted using the Review Manager (RevMan version 5.3, the Nordic Cochrane Center, the Cochrane Collaboration, 2014) and Stata software (version 14; StataCorp LLC, College Station, TX, United States).

## Result

### Literature search

**Figure 1:**
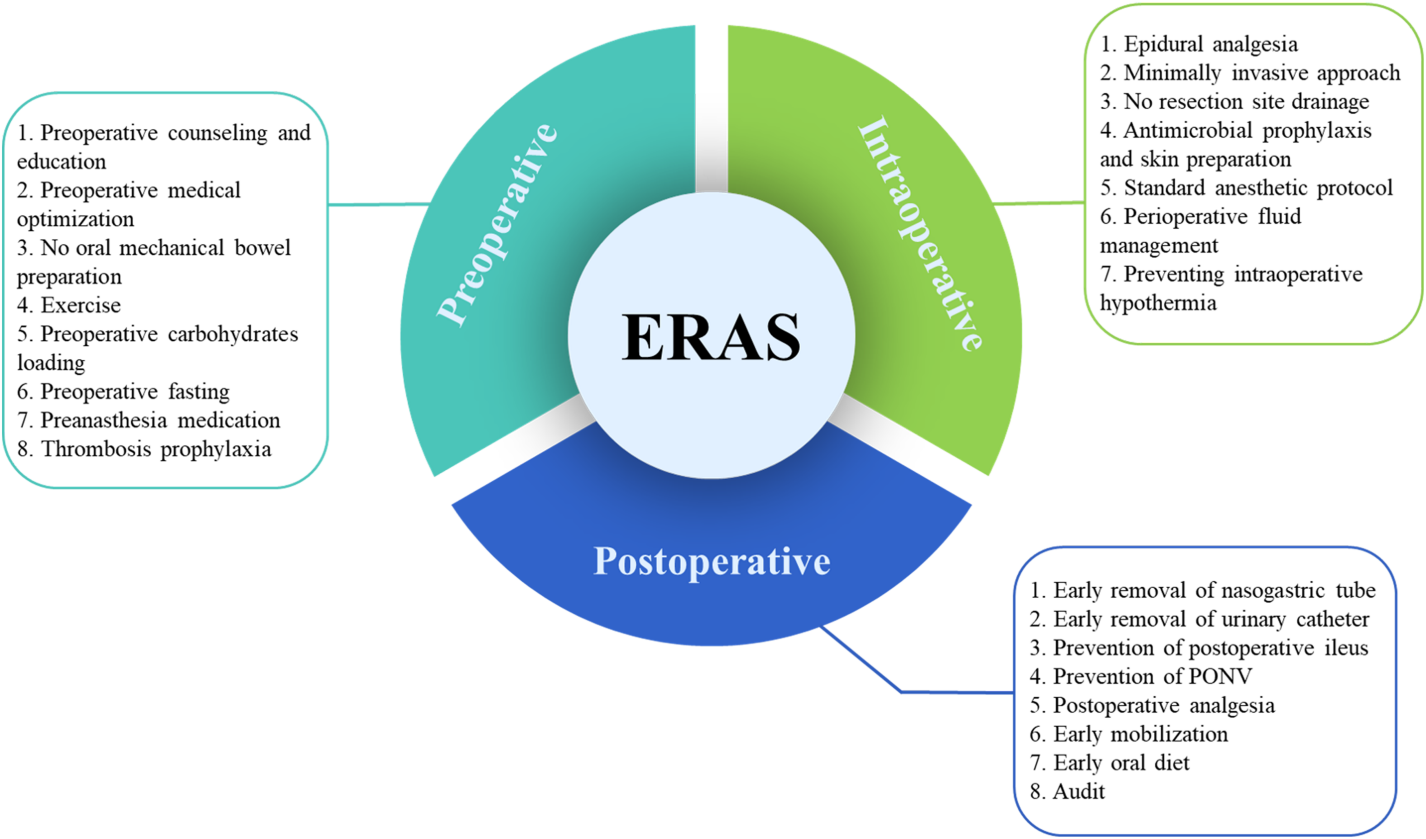
Care elements implemented in the ERAS program for radical cystectomy. ERAS, enhanced recovery after surgery; PONV, postoperative nausea and vomiting.

**Figure 2:**
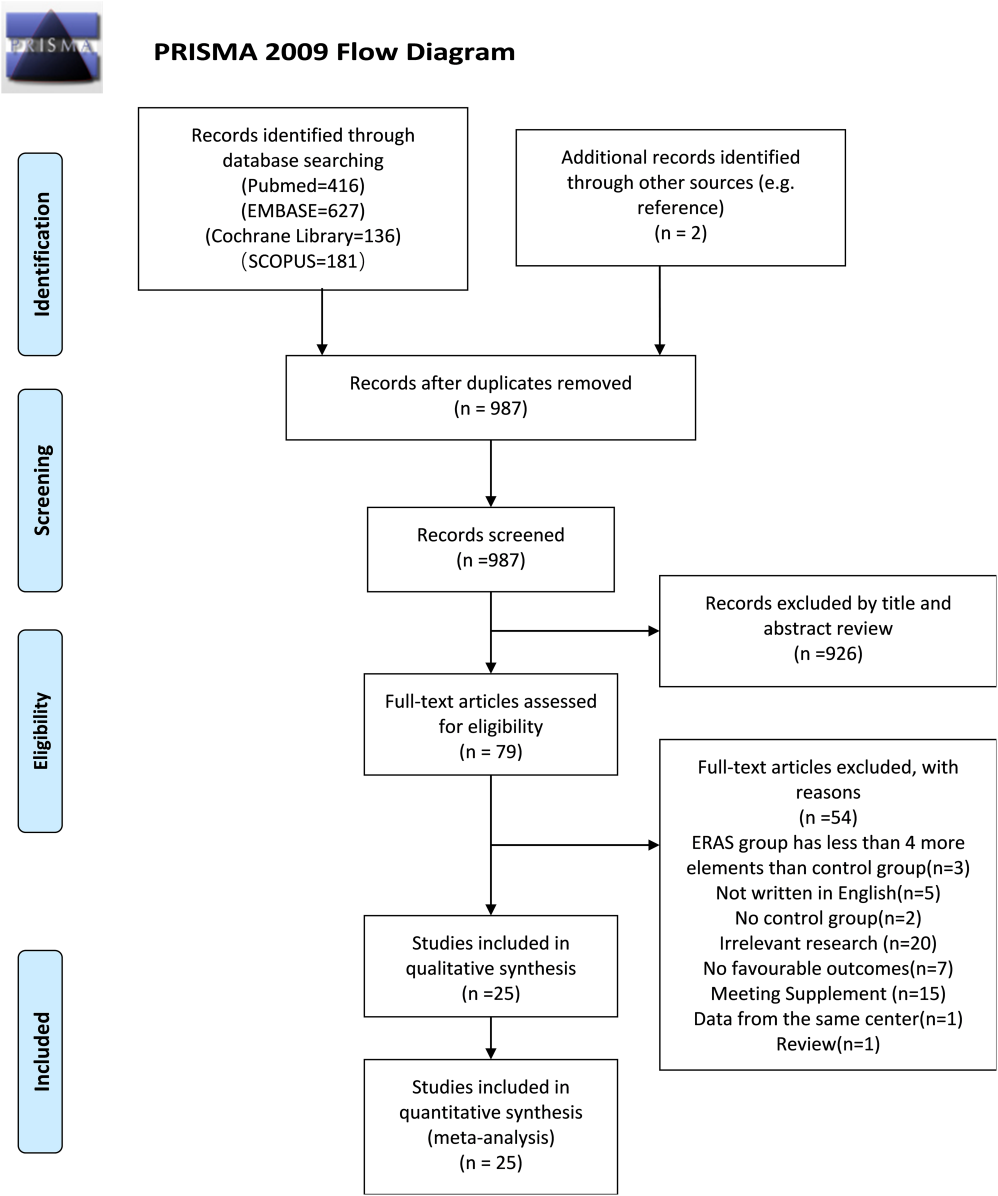
PRISMA flow diagram of literature retrieval. PRISMA, Preferred Reporting Items for Systematic Reviews and Meta-Analyses.

Care elements implemented in the ERAS program for radical cystectomy was shown in Figure 1. A flow diagram indicating the search procedures is presented in Figure 2. A total of 1,360 potential articles were distinguished, including 416 PubMed citations, 627 EMBASE citations, 181 SCOPUS citations, and 136 Cochrane Library citations. Furthermore, a manual search of the reference lists also yielded two relevant studies. After checking for duplicates and reviewing titles, abstracts, and full texts, 25 eligible articles were included in the qualitative assessment (15–39).

### Characteristics of the included studies

Tables 1–3 summarize the baseline characteristics and major perioperative outcomes. The study included 20 cohort studies (15, 17–19, 21, 23–37) and five RCTs (16, 20, 22, 38, 39). The publication dates of the included articles ranged from 2013 to 2022. All eligible articles were written in English.

**Table 1:** Baseline characteristics of included studies.

Study	Country	Period	Study type	Sample size			Age		Gender (male ratio)		ERAS elements		Surgery type, *n* (%) (open/RARC/lap)		Urinary diversion type, *n* (%) (ONE/IC/UR)	
				Total	ERAS	Con.	ERAS	Con.	ERAS	Con.	ERAS	Con.	ERAS	Con.	ERAS	Con.
Zhang et al.	China	2014−2018	RCS	443	185	258	63.0 ± 9.9	62.6 ± 10.6	171 (92%)	240 (93%)	18	8	NR	NR	52/114/19 (28.1/61.6/10.3)	62/163/33 (24/63.2/12.8)
Vlad et al.	Romania	2017	RCT	90	45	45	62.1 ± 6.2	63 ± 6.7	40 (89%)	39 (88%)	16	12	NR	NR	13/32/0 (28.9/71.1/0)	10/35/0 (22.2/77.8/0)
Mukhtar et al.	U.K.	2007–2012	PCS	77	51	26	67.7 ± 7.8	69.8 ± 8.3	39 (76%)	21 (81%)	14	3	51/0/0 (100/0/0)	26/0/0 (100/0/0)	3/48/0 (5.9/94.1/0)	2/24/0 (7.7/92.3/0)
Romagnoli et al.	Italy	2016–2017	ACS	40	20	20	70 [60–76]	72 [66–75]	16 (80%)	15 (75%)	14	6	19/0/1 (95/0/5)	20/0/0 (100/0/0)	9/11/0 (45/55/0)	8/12/0 (40/60/0)
Pramod et al.	Indonesia	2018–2019	RCS	21	9	12	68.9 no SD	67.5 no SD	7 (75%)	9 (75%)	14	4	NR	NR	0/0/9 (0/0/100)	0/0/12 (0/0/100)
Pang et al.	U.K.	2007–2016	PCS	453	393	60	71 [65.0–76.0]	66 [60.8–70.3]	303 (77%)	52 (87%)	23	6	NR	NR	25/368/0 (6/94/0)	25/35/0 (42/58/0)
Palumbo et al.	Italy	2013–2016	PCS	114	74	40	71.7 ± 11.3	73.9 ± 10.3	59 (80%)	32 (80%)	13	5	74/0/0 (100/0/0)	40/0/0 (100/0/0)	7/17/25 (9.5/23/33.8)	1/12/14 (2.5/30/35)
Lin et al.	China	2014–2016	RCT	290	145	145	62.9 ± 10.1	63.3 ± 10.3	124 (86%)	126 (87%)	11	5	25/7/112 (17.4/4.8/77.8)	34/8/103 (23.4/5.5/71.1)	53/91/0 (36.8/63.2/0)	56/89/0 (38.6/61.4/0)
Frees et al.	Canada	NR	RCT	23	10	13	65.8 (49–86)	70.4 (51–84)	7 (70%)	11 (85%)	11	7	NR	NR	3/7/0 (30/70/0)	2/11/0 (15.4/84.6/0)
Collins et al.	Sweden	2003–2014	PCS	221	135	86	70 [63–74]	66 [59–71]	101 (75%)	71 (83%)	18	4	0/135/0 (0/100/0)	0/86/0 (0/100/0)	38/97/0 (28.1/71.9/0)	48/38/0 (55.8/44.2)
Cerruto et al.	Italy	2010–2011	ACS	22	9	13	61.2 ± 10.6	67.1 ± 5.5	9 (100%)	12 (92%)	13	6	NR	NR	0/9/0 (0/100/0)	0/13/0 (0/100/0)
Kukreja et al.	United States	2011–2015	ACS	200	79	121	70.6 [65.2–77.7]	69.5 [61.9–77]	49 (62%)	99 (81.8%)	14	2	35/44/0 (44.3/55.7.0)	57/64/0 (52.9/47.1/0)	5/71/0 (6.3/89.9/0)	7/113/0 (5.8/93.4/0)
Persson et al.	Sweden	2010–2011	ACS	70	31	39	67 (42–80)	66 (53–80)	21 (68%)	30 (77%)	17	12	0/31/0 (0/100/0)	0/39/0 (0/100/0)	5/26/0 (17/83/0)	13/26/0 (33/67/0)
Liu et al.	Canada	2007–2016	RCS	260	84	176	68.9 no SD	67.84 no SD	127 (72.2%)	69 (82.1%)	13	3	NR	NR	0/84/0 (0/100/0)	0/176/0 (0/100/0)
Guleser et al.	Turkey	2017–2020	RCS	46	18	28	66.9 ± 8.01	63.32 ± 8.02	25 (89%)	25 (89%)	13	3	NR	NR	0/18/0 (0/100/0)	0/28/0 (0/100/0)
Wei et al.	China	2010–2017	RCS	192	91	101	69.3 (52–84)	67.4 (48–81)	85 (93.4%)	93 (92.1%)	13	3	0/0/91 (0/0/100)	NR	3/82/6 (3.3/90.1/6.6)	2/91/8 (2/90.1/7.9)
Semerjian et al.	United States	2015–2016	ACS	110	56	54	68.6 [55.9–81.3]	69.5 [60.5–78.5]	48 (86%)	47 (87%)	12	7	48/8/0 (86/14/0)	52/2/0 (96/4/0)	3/53/0 (5/95/0)	3/51/0 (6/94/0)
Hanna et al.	United States	2010–2018	RCS	296	150	146	69 [62–75]	69 [62–76]	116 (77.3%)	120 (82.2%)	14	4	105/45/0 (70/30/0)	102/44/0 (69.9/44/0)	36/110/0 (24/73.3/0)	39/103/0 (26.7/70.5/0)
Dunkman et al.	United States	2015–2017	ACS	200	100	100	66 [61–72.25]	68 [69.75–77]	69 (69%)	79 (79%)	15	5	NR	NR	NR	NR
Brockman et al.	United States	2012–2015	ACS	299	152	147	68 no SD	66.7 no SD	NR	NR	11	3	96/56/0 (63.2/36/8/0)	114/33/0 (77.6/22/4/0)	32/120/0 (21.1/63.2/0)	30/117/0 (20.4/79.6/0)
Jensen et al.	Denmark	2011–2013	RCT	107	50	57	69 (46–85)	71 (47–91)	39 (78%)	40 (70%)	14	4	41/9/0 (82/18/0)	44/13/0 (77/23/0)	5/44/1 (10/88/2)	7/48/2 (12/84/4)
Saar et al.	Germany	2007–2010	PCS	62	31	31	67.2 ± 10.2	61.6 ± 12.6	27 (87.1%)	27 (87.1%)	8	3	0/31/0 (0/100/0)	0/31/0 (0/100/0)	8/23/0 (25.8/74.2/0)	12/19/0 (38.7/61.3/0)
Lannes et al.	France	2015–2019	RCS	150	76	74	71 (42–87)	69 (32–91)	60 (78.9%)	54 (73%)	22	4	19/57/0 (25/75/0)	54/20/0 (73/27/0)	46/28/0 (60.6/36.8/0)	38/34/2 (51.3/46/2.7)
Olaru et al.	Romania	NR	RCT	20	10	10	62.5 no SD	62.0 no SD	10 (100%)	10 (100%)	13	1	NR	NR	6/4/0 (60/40/0)	5/5/0 (50/50/0)
Llorente et al.	Spanish	2014–2017	PCS	277	147	130	70.39 ± 8.29	68.4 ± 9.41	127 (86.4%)	108 (83.1%)	17	8	140/1/6 (95.2/0.7/4.1)	127/0/3 (97.7/0/2.3)	19/112/0 (13/76.7/0)	17/95/0 (13.1/73.1/0)

ERAS, enhanced recovery after surgery; Con., control; RCT, randomized controlled trial; PCS, prospective cohort study; RCS, retrospective cohort study; ACS, ambispective cohort study; Open, open radical cystectomy; RARC: robot-assisted radical cystectomy; Lap, laparoscopic radical cystectomy; ONE, orthotopic neobladder; IC, ileal conduit; UR, ureterostomy; NR, not reported; [ ] = interquartile range; ( ) = range; mean ± standard deviation (SD).

**Table 2:** Outcomes of continuous variables in included studies.

Studies	LOS, day		Flatus, day		Stool, day		Normal diet, day		Intraoperative blood loss, ml		Operative time, min		Mobilization, day	
	ERAS	Con.	ERAS	Con.	ERAS	Con.	ERAS	Con.	ERAS	Con.	ERAS	Con.	ERAS	Con.
Zhang et al.^a^	8.2	11.6	0.35	1.3	NR	NR	NR	NR	342 (200–1,500)	468 (300–2,600)	308 (217–420)	314 (211–436)	0.4	1.19
Vlad et al.	16 [15–17]	18 [16.5–21]	1 [1–2]	5 [3–6]	2 [2–3.5]	5 [4–7]	5 [3–6]	6 [4–7]	NR	NR	322 [303–369]	331 [307–366]	NR	NR
Mukhtar et al.	4 (6–62)	11.5 (7–24)	4.6 ± 0.2	6.2 ± 0.4	6.1 ± 0.3	7.4 ± 0.5	4.6 ± 0.2	5.9 ± 0.3	NR	NR	NR	NR	NR	NR
Romagnoli et al.	10 [8–12]	13 [11–14]	1.5 [1–2.8]	3 [2–3.8]	4 [3–5.8]	6 [4.5–6]	NR	NR	NR	NR	260 ± 56	293 ± 54	1.8 ± 1.0	8.8 ± 2.2
Pramod et al.	4.77 ± 1.2	10 ± 4.767	NR	NR	NR	NR	NR	NR	1,200 ± 627	1,583 ± 722	NR	NR	NR	NR
Pang et al.	8 [6–13]	18 [13–25]	NR	NR	NR	NR	NR	NR	600 [383–969]	1,050 [900–1,575]	NR	NR	NR	NR
Palumbo et al.	13.1 ± 3.9	16.5 ± 6.2	2.4 ± 1	3.6 ± 1.7	3.6 ± 1.6	6.0 ± 2.0	1.0 ± 0	6 ± 1.7	620.2 ± 453.4	743.8 ± 405.1	267.9 ± 60.2	290.9 ± 63.7	1.0 ± 0.1	1.5 ± 0.6
Lin et al.	NR	NR	2.4 [1.5–3.3]	2.9 [2–3.8]	NR	NR	5.2 [4–8]	7 [4.8–9]	NR	NR	310.8 ± 101	300.4 ± 88.9	2.7 [1.7–4]	3 [2–4.1]
Frees et al.	6.1 (5–7)	7.39 (5–11)	2.5 (2–4)	3.6 (2–5)	4.3 (2–6)	6.3 (3–9)	NR	NR	727.3 (300–1,500)	900 (400–2,500)	269.3 (210–350)	253.3 (210–340)	NR	NR
Collins et al.	8 [6–10]	9 [8–13]	NR	NR	NR	NR	NR	NR	NR	NR	NR	NR	NR	NR
Cerruto et al.	15 [14 −15]	15 [15–16]	2 [1–3]	4 [4–5]	4 [3–4]	5 [5–7]	NR	NR	600.2 ± 206.2	769.2 ± 370.6	250.6 ± 18.6	259.6 ± 48.5	0.8 ± 0.3	1.1 ± 0.3
Kukreja et al.	5 [4–7]	8 [6–13]	NR	NR	NR	NR	3 [2–3]	5 [4–7]	NR	NR	432 [384–510]	408 [354–492]	NR	NR
Persson et al.	11 (9–107)	12 (10–64)	NR	NR	3.7	5.4	NR	NR	1,100	1,000	349	355	NR	NR
Liu et al.	10.9 ± 8.6	14.3 ± 14.6	NR	NR	NR	NR	NR	NR	NR	NR	NR	NR	NR	NR
Guleser et al.	10.4 ± 4.6	14.8 ± 6.4	3.0 ± 1.8	4.4 ± 2.4	NR	NR	NR	NR	NR	NR	NR	NR	NR	NR
Wei et al.	4.8 ± 1.7	11.2 ± 2.7	0.4 ± 0.3	1.3 ± 0.8	0.7 ± 0.2	3.4 ± 1.5	NR	NR	368 (245–1,800)	425 (330–2,600)	303 (240–420)	316 (210–480)	0.4 ± 0.1	3.1 ± 0.2
Semerjian et al.^a^	5	8.5	NR	NR	NR	NR	NR	NR	650	725	263	261	NR	NR
Hanna et al.	8 [7–11.5]	8 [6–11]	NR	NR	NR	NR	5 [4.5–8]	6 [4–7]	500 [400–925]	600 [400–1,000]	369 [311–444]	384 [327–483]	1 [1–2]	2 [1–2]
Dunkman et al.	10 ± 7.03	14.86 ± 11.05	4.0 ± 1.9	5.8 ± 3.8	NR	NR	3.2 ± 2.5	9.7 ± 8.8	999.3 ± 549.9	1,411.3 ± 902	NR	NR	1.3 ± 0.8	2.0 ± 1.2
Brockman et al.^a^	7.1	10	NR	NR	NR	NR	NR	NR	NR	NR	439.4	413.5	NR	NR
Jensen et al.^a^	8 (3–30)	8 (4–55)	1.0	2.0	4.0	4.0	NR	NR	NR	NR	NR	NR	NR	NR
Saar et al.	18 ± 5.1	18.1 ± 6.3	1.9 ± 0.8	2.4 ± 1.3	NR	NR	4 ± 2.2	6.6 ± 3.5	383 ± 203	424 ± 182	405.6 ± 78	415.2 ± 78	0.7 ± 0.4	1.3 ± 0.7
Lannes et al.	12.7 ± 6.2	13.1 ± 5.7	4.5 ± 2.2	5.5 ± 2.6	NR	NR	NR	NR	467.5 (50–2,800)	576.5 (100–2,000)	414.3 (180–663)	378.4 (240–660)	NR	NR
Olaru et al.^a^	NR	NR	2.5	4.0	6.0	7.0	4.5	6.0	NR	NR	350.0	354.0	NR	NR
Llorente et al.^a^	14	12.5	NR	NR	NR	NR	NR	NR	500 [300–700]	600 [369–1,000]	275 [215–350]	290 [231–345]	NR	NR

ERAS, enhanced recovery after surgery; Con., control; LOS, length of stay; Flatus, time to first flatus; Stool, time to passage of first stool; NR, not reported; [ ] = interquartile range; ()=range; mean ± standard deviation (SD).

^a^ Data presented as mean without SD.

**Table 3:** Outcomes of categorical variables in included studies.

Studies	Overall complications, *n* (%)		Postoperative ileus, *n* (%)		Readmission, *n* (%)		Mortality, *n* (%)		Urine leakage, *n* (%)		Transfusion rate, *n* (%)	
	ERAS	Con.	ERAS	Con.	ERAS	Con.	ERAS	Con.	ERAS	Con.	ERAS	Con.
Zhang et al.	31 (16.8)	82 (31.8)	4 (2.2)	12 (4.7)	24 (13.0)	72 (27.9)	3 (1.6)	4 (1.6)	4 (2.2)	14 (5.4)	24 (13.0)	72 (27.9)
Vlad et al.	21 (46.6)	26 (57.8)	15 (33.3)	21 (53.3)	3 (6.6)	5 (11.1)	0	2 (4.4)	1 (2.2)	1 (2.2)	NR	NR
Mukhtar et al.	20 (39.2)	12 (43.1)	3 (5.9)	0	0	0	NR	NR	NR	NR	NR	NR
Romagnoli et al.	6 (20)	3 (15)	5 (25)	1 (5)	1 (5)	3 (15)	NR	NR	NR	NR	5 (25)	8 (40)
Pramod et al.	NR	NR	NR	NR	0	1 (8.3)	NR	NR	NR	NR	5 (55.6)	11 (91.7)
Pang et al.	NR	NR	NR	NR	59 (15)	15 (25)	8 (2)	3 (5)	NR	NR	32 (8.1)	15 (25)
Palumbo et al.	35 (47.3)	25 (62.5)	7 (9.5)	5 (12.5)	7 (9.5)	6 (15)	NR	NR	NR	NR	19 (25.7)	16 (40)
Lin et al.	55 (38.2)	55 (37.9)	20 (13.9)	20 (13.8)	5 (3.5)	5 (3.4)	0	0	3 (2.1)	3 (2.1)	NR	NR
Frees et al.	NR	NR	1 (10)	0	1 (10)	0	NR	NR	NR	NR	NR	NR
Collins et al.	77 (57)	51 (59.3)	NR	NR	44 (32.6)	25 (29.1)	3 (2.2)	2 (2.3)	NR	NR	NR	NR
Cerruto et al.	9 (100)	13 (100)	NR	NR	0	0	NR	NR	NR	NR	1 (11.1)	6 (46.2)
Kukreja et al.	56 (70.9)	99 (81.8)	24 (30.4)	65 (53.7)	24 (30.4)	34 (28.1)	5 (6.3)	10 (8.3)	6 (7.6)	6 (5)	40 (50.6)	52 (43)
Persson et al.	14 (45.2)	23 (59)	5 (16.1)	13 (33.3)	1 (3.2)	10 (25.6)	NR	NR	1 (3.2)	0	NR	NR
Liu et al.	39 (46.4)	91 (51.7)	17 (20.2)	49 (27.8)	16 (19)	35 (19.9)	NR	NR	NR	NR	NR	NR
Guleser et al.	NR	NR	3 (16.7)	15 (25)	NR	NR	NR	NR	NR	NR	NR	NR
Wei et al.	14 (15.4)	29 (28.7)	4 (4.4)	7 (6.9)	4 (4.4)	11 (10.9)	3 (3.3)	4 (4)	1 (1.1)	2 (2)	4 (4.4)	15 (14.9)
Semerjian et al.	NR	NR	18 (33)	24 (44)	11 (19)	8 (14.8)	NR	NR	NR	NR	NR	NR
Hanna et al.	95 (63.3)	91 (62.3)	44 (29.3)	31 (21.2)	54 (36)	57 (39)	NR	NR	NR	NR	NR	NR
Dunkman et al.	NR	NR	36 (36)	65 (65)	19 (19)	38 (38)	2 (2)	2 (2)	NR	NR	5 (5)	10 (10)
Brockman et al.	91 (59.9)	86 (58.5)	19 (12.8)	17 (11.9)	47 (30.9)	42 (28.6)	NR	NR	NR	NR	70 (46.1)	91 (61.9)
Jensen et al.	3 (6)	4 (7)	NR	NR	NR	NR	50 (100)	57 (100)	NR	NR	NR	NR
Saar et al.	12 (38.7)	15 (48.4)	NR	NR	2 (6.5)	6 (19.4)	2 (6.5)	0	2 (6.5)	0	NR	NR
Lannes et al.	NR	NR	12 (15.8)	18 (24.3)	21 (27.6)	26 (35.1)	NR	NR	NR	NR	13 (17.1)	26 (35.1)
Olaru et al.	4 (40)	6 (60)	2 (20)	4 (40)	NR	NR	NR	NR	1 (10)	0	NR	NR
Llorente et al.	97 (66)	92 (70.5)	NR	NR	51 (34.6)	48 (36.7)	3 (2)	7 (5.4)	NR	NR	39 (26.5)	49 (37.7)

ERAS, enhanced recovery after surgery; Con., control; Mortality: 90-day mortality; NR, not reported; [ ] = interquartile range; () = range; mean ± standard deviation (SD).

### Patient characteristics

Through layers of selection, 4,083 patients were finally enrolled in our meta-analysis. The detailed characteristics of the participant are shown in Table 1. A total of 2,151 (52.7%) and 1,932 (47.3%) patients were enrolled in the ERAS and control groups, respectively.

### ERAS elements

Elaborate details of ERAS elements evaluated in each study are summarized in Tables 4A–C. The number of ERAS elements concluded in the ERAS and control groups ranged from 8 to 23 and 1 to 12, respectively. The element of ERAS was adopted from the guideline and an improved study that demonstrated the benefits of exercise (22). The most used element was early oral diet (all studies were adopted), followed by early mobilization (adopted by 23 studies). Although the ERAS elements were various in the included studies, the overlapping parts are shown in Tables 4A–C.

**Table 4A:** Detailed ERAS elements of included studies (I).

ERAS elements	Studies								
	Zhang	Vlad	Mukhtar	Romagnoli	Pramod	Pang	Palumbo	Lin	Frees
Preoperative interventions									
Preoperative counseling and education	√	√	√	√		√	√	√^a^	√^a^
Preoperative medical optimization	√^a^	√	–		–	√^a^			√
No oral mechanical bowel preparation		√^a^	√^a^	√^a^	√^a^	√^a^	√^a^	√^a^	√
Exercise					√^a^	√^a^			
Preoperative carbohydrates loading	√^a^	√	√^a^		√^a^	√^a^			
Preoperative fasting	√	√		√	√	√	√	√	√
Preanasthesia medication		√	–			√^a^	√	√^a^	
Thrombosis prophylaxis	√^a^	√		√^a^	–	√^a^	√^a^		√
Intraoperative interventions									
Epidural analgesia	√	√	√^a^	√^a^	√^a^	√^a^			√
Minimally invasive approach	√^a^		√^a^			√			
No resection site drainage	√^a^					√^a^			
Antimicrobial prophylaxis and skin preparation	√	√		√	√^a^	√^a^	√^a^	√	
Standard anesthetic protocol	√	√	√	√	√	√	√		
Perioperative fluid management	√^a^	√	√^a^	√	√	√	√^a^	√^a^	√^a^
Preventing intraoperative hypothermia	√		√^a^			√^a^	√^a^		
Postoperative interventions									
Early removal of nasogastric tube	√^a^	√^a^	√^a^	√^a^	√^a^	√^a^	√^a^	√^a^	
Early removal of urinary catheter	–		√^a^	√^a^		√^a^			
Prevention of postoperative ileus	√	√^a^		√	√^a^	√^a^			√^a^
Prevention of PONV	√^a^		√^a^	√^a^	√^a^	√^a^			√^a^
Postoperative analgesia	√	√	√^a^		√	√	√	√	
Early mobilization	√^a^	√	√	√^a^	√^a^	√^a^	√^a^	√	
Early oral diet	√^a^	√^a^	√^a^	√^a^	√^a^	√^a^	√^a^	√	√
Audit						√^a^		√^a^	√

**Table 4B:** Detailed ERAS elements of included studies (II).

ERAS elements	Studies								
	Collins	Cerruto	Kukreja	Persson	Liu	Guleser	Wei	Semerjian	Hanna
Preoperative interventions									
Preoperative counseling and education	√^a^	√	√	√^a^	√		√	√	√
Preoperative medical optimization	√^a^		√^a^		√^a^	√^a^	√^a^		√^a^
No oral mechanical bowel preparation	√^a^		√^a^	√	√^a^	√^a^	√^a^	√^a^	√^a^
Exercise									
Preoperative carbohydrates loading	√^a^		√^a^	√^a^		√^a^		√^a^	
Preoperative fasting	√	√		√	√	√		√	√
Preanasthesia medication	√^a^			√		√^a^			
Thrombosis prophylaxis	√^a^	√	√^a^	√	√^a^	√^a^	√^a^		√^a^
Intraoperative interventions									
Epidural analgesia		√^a^		√	√^a^	√^a^	√^a^	√^a^	√
Minimally invasive approach	√					√	√		
No resection site drainage				√^a^					
Antimicrobial prophylaxis and skin preparation	√^a^	√	√^a^	√	√^a^	√^a^	√^a^		√^a^
Standard anesthetic protocol	√		√			√		√	√
Perioperative fluid management	√^a^	√	√^a^	√	√^a^		√^a^	√^a^	√^a^
Preventing intraoperative hypothermia	√^a^	√^a^	√^a^	√					√^a^
Postoperative interventions									
Early removal of nasogastric tube	√^a^	√^a^	√^a^	√	√^a^	√^a^		√	√^a^
Early removal of urinary catheter									
Prevention of postoperative ileus		√	√^a^				√^a^		
Prevention of PONV	√^a^	√^a^	√^a^	√			√^a^	√	√^a^
Postoperative analgesia	√	√^a^		√	√		√	√^a^	
Early mobilization	√^a^	√^a^	√^a^	√^a^	√^a^	√^a^	√^a^	√	√^a^
Early oral diet	√^a^	√^a^	√^a^	√^a^	√^a^	√^a^	√^a^	√	√^a^
Audit	√^a^			√	√^a^				

**Table 4C:** Detailed ERAS elements of included studies (III).

ERAS elements	Studies						
	Dunkman	Brockman	Jensen	Saar	Lannes	Olaru	Llorente
Preoperative interventions							
Preoperative counseling and education	√	√	√		√^a^	√^a^	√
Preoperative medical optimization		√^a^	√^a^		√^a^	√^a^	√^a^
No oral mechanical bowel preparation	√^a^				√^a^	√^a^	√
Exercise			√^a^				√^a^
Preoperative carbohydrates loading	√^a^			√^a^	√^a^	√^a^	√^a^
Preoperative fasting	√	√	√		√	√	√^a^
Preanasthesia medication	√^a^				√^a^	√^a^	√
Thrombosis prophylaxis	√^a^	√^a^	√^a^		√^a^	√^a^	√
Intraoperative interventions							
Epidural analgesia	√	√^a^			√^a^	√^a^	
Minimally invasive approach			√		√		
No resection site drainage				√^a^	√^a^		
Antimicrobial prophylaxis and skin preparation	√^a^	√^a^	√^a^	√	√^a^	√^a^	√
Standard anesthetic protocol	√	√	√		√		
Perioperative fluid management	√^a^	√^a^			√^a^		√^a^
Preventing intraoperative hypothermia		√^a^			√^a^		√
Postoperative interventions							
Early removal of nasogastric tube		√^a^		√^a^	√^a^	√^a^	√^a^
Early removal of urinary catheter			√^a^		√^a^		
Prevention of postoperative ileus	√^a^			√^a^	√^a^	√^a^	√^a^
Prevention of PONV	√^a^		√^a^		√^a^		√
Postoperative analgesia	√		√^a^	√	√		√
Early mobilization	√^a^		√^a^	√	√^a^	√^a^	√^a^
Early oral diet	√^a^	√^a^	√^a^	√^a^	√^a^	√^a^	√^a^
Audit			√^a^		√^a^		

ERAS, enhanced recovery after surgery; PONV, postoperative nausea and vomiting.

^a^ Included in the ERAS group but not in the control group.

### Quality assessment

The quality assessment of the included studies is presented in Tables 5A,B. Finally, 20 cohort studies received a NOS score ≥6. As for RCTs, only one study was double-blinded (16), and the other four studies had at least one unclear bias (20, 22, 38, 39), as shown in Supplementary Figure S2.

**Table 5A:** Detailed quality assessment of cohort studies (I).

Items of NOS	Studies										
	Zhang	Mukhtar	Romagnoli	Pramod	Pang	Palumbo	Collins	Cerruto	Kukreja	Persson	Liu
Selection											
Representativeness of the exposed cohort	*	*	*		*	*	*	*	*	*	*
Selection of the non-exposed cohort	*	*	*	*	*	*	*	*	*	*	*
Ascertainment of exposure	*	*	*	*	*	*	*	*	*	*	*
Demonstration that outcome of interest was not present at start of study	*	*	*	*	*	*	*	*	*	*	*
Comparability											
Comparability of cohorts on basis of the design or analysis	**	**	**	*	*	**	*	**	**	**	**
Outcome											
Assessment of outcome	*	*	*	*	*	*	*	*	*	*	*
Was followed up long enough for outcomes to occur	*		*	*	*	*	*	*	*	*	*
Adequacy of follow-up of cohorts	*										
Total	9	7	8	6	7	8	7	8	8	8	8

**Table 5B:** Detailed quality assessment of cohort studies (II).

Items of NOS	Studies								
	Guleser	Wei	Semerjian	Hanna	Dunkman	Brockman	Saar	Lannes	Llorente
Selection									
Representativeness of the exposed cohort	*	*	*	*	*	*	*	*	*
Selection of the non-exposed cohort	*	*	*	*	*	*	*	*	*
Ascertainment of exposure	*	*	*	*	*	*	*	*	*
Demonstration that outcome of interest was not present at start of study	*	*		*		*	*		*
Comparability									
Comparability of cohorts on basis of the design or analysis	**	**	**	**	*	*	**	**	**
Outcome									
Assessment of outcome	*	*	*	*	*	*	*	*	*
Was followed up long enough for outcomes to occur		*			*	*			*
Adequacy of follow-up of cohorts									
Total	7	8	7	8	6	7	7	6	8

NOS, Newcastle–Ottawa Scale.

A study can be awarded one star for each numbered item within the selection and outcome categories. A maximum of two stars can be given for comparability. Study rates ≥6 are eligible.

### Effect of ERAS on the outcomes

#### Length of stay

**Figure 3:**
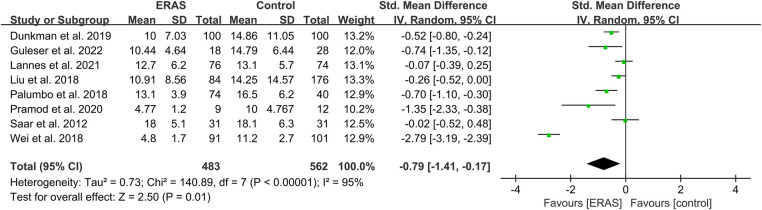
Meta-analysis of length of stay (LOS) between the ERAS and control group. ERAS, enhanced recovery after surgery; CI, confidence interval.

A total of eight studies reported the length of stay (17, 18, 24, 27–30, 37), and the pooled analysis of meta-analysis indicated that patients had a significantly shorter length of stay in the ERAS group (SMD = −0.79; 95% CI: −1.41 to −0.17; *P* = 0.01) with significantly high heterogeneity (*I*^2 ^= 95%; *P* < 0.00001) compared with that of the control group, as shown in Figure 3. No publication bias was found using Egger's test (*P* = 0.486).

#### Time to first flatus and stool

**Figure 4:**
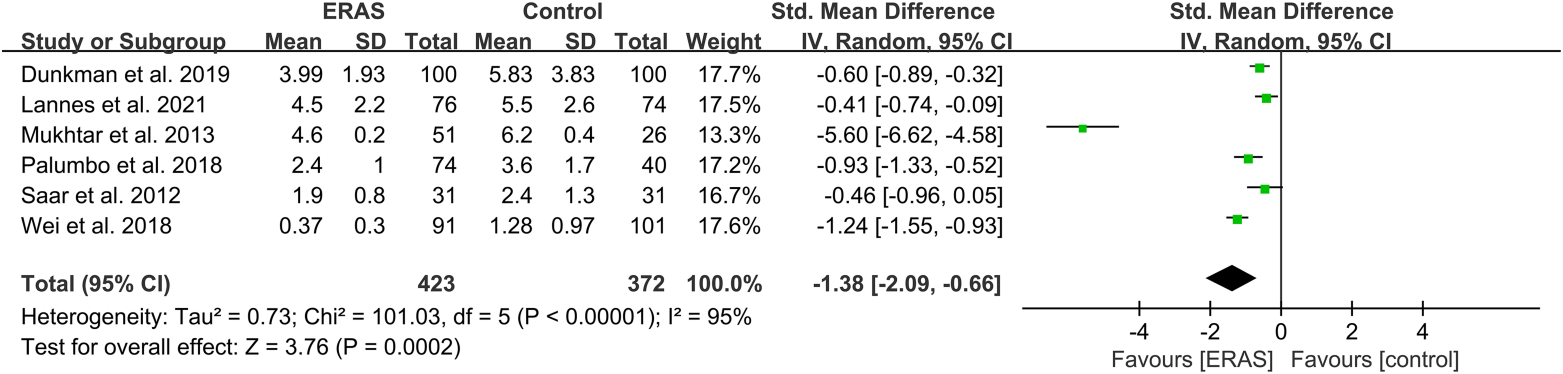
Meta-analysis of flatus time between the ERAS and control group. ERAS, enhanced recovery after surgery; CI, confidence interval.

A total of 14 studies reported the time to first flatus (16, 20–22, 24, 25, 27, 29, 30, 35–39), and 11 studies reported the time to first stool (17, 20, 22, 25, 26, 29, 35–39). Among the studies of time to first stool, only four presented the data in the format of mean ± SD (17, 29, 36, 37). Therefore, we performed a qualitative analysis rather than a meta-analysis. Among the 11 studies that reported the time to first stool, eight indicated that the ERAS group had a significantly shorter time to defecation (17, 20, 26, 29, 35–38), while the other three showed that no difference was found (22, 25, 39), as shown in Table 2. For the analysis of time to first flatus, the pooled data of six eligible studies indicated that participants in the ERAS group had a significantly shorter time to flatus (SMD = −1.38; 95% CI: −2.09 to −0.66; *P* = 0.0002) with high heterogeneity (*I*^2 ^= 95%; *P* < 0.00001) between studies (Figure 4). No publication bias was found using Egger's test (*P* = 0.092).

#### Time to normal diet and mobilization

A qualitative analysis was performed for the time to normal diet and mobilization since the available studies for mean ± SD were less than or equal to 5. Of the nine studies that reported the time to normal diet (16, 20, 27, 30, 31, 34, 36, 37, 39), eight indicated that the ERAS group had a significantly shorter time to normal diet, and one did not mention the *P*-value between the two groups. Moreover, the ERAS group showed early mobilization in studies.

#### Intraoperative blood loss and operative time

**Figure 5:**
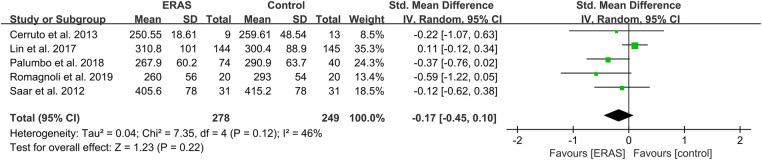
Meta-analysis of operation duration between the ERAS and control group. ERAS, enhanced recovery after surgery; CI, confidence interval.

A total of 14 studies reported the intraoperative blood loss (15, 18, 21, 23–27, 29, 30, 33, 34, 37, 38), and 16 studies reported the operative time (15, 16, 19–21, 23–27, 29, 31, 34, 35, 37–39). However, only five studies presented IBS in mean ± SD format, and there were not enough studies after identifying no difference in surgical approach. Therefore, we conducted a qualitative analysis of IBS. Among the 14 studies, two showed a significant reduction of IBS in the case of excluding surgical differences (23, 37). Moreover, a meta-analysis of operative time showed no significant difference between the ERAS and control groups, as shown in Figure 5.

#### Postoperative complications

**Figure 6:**
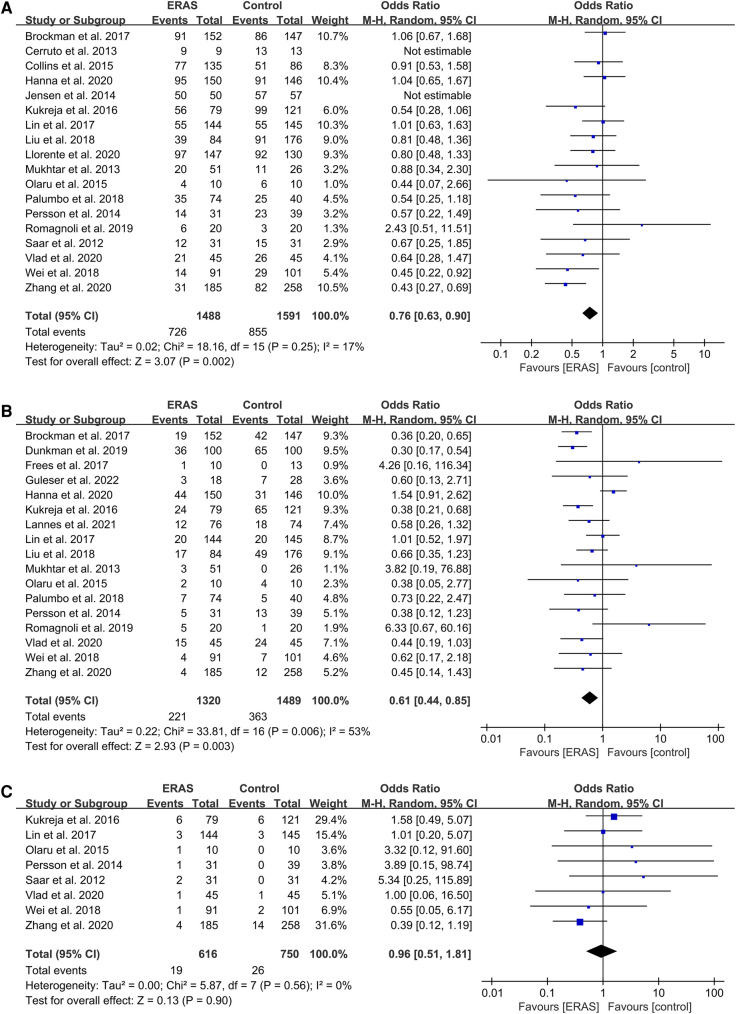
Meta-analysis of postoperative complications between the ERAS and control group. (A) Overall complication; (B) Intestinal obstruction; (C) Urine leakage. ERAS, enhanced recovery after surgery; CI, confidence interval.

Of the 18 studies that reported on overall complications (16, 19–23, 25–29, 31, 32, 34–37, 39), three reported that the ERAS group had decreased rates of overall postoperative complications (21, 25, 29). Other studies found no significant difference between the two groups. The pooled OR of all 18 studies was 0.76 (95% CI: 0.63–0.90; *P* = 0.002) with low heterogeneity by random effects, which significantly reduced the overall complications rate in the ERAS group, as shown in Figure 6A.

The pooled OR of 17 studies about postoperative ileus (16, 17, 19–21, 24, 26, 28–31, 34–39) was 0.61 (95% CI: 0.44–0.85; *P* = 0.003) with moderate heterogeneity (*I*^2 ^= 53%; *P* = 0.006) by random effects, which indicated a significant reduction of POI in the ERAS group compared with the control group, as shown in Figure 6B.

We did not find any significant differences in the urine leakage complications (16, 18, 20, 21, 26, 29, 31, 39), with an OR of 0.96 (95% CI: 0.51–1.81; *P* = 0.90) and low heterogeneity (*I*^2 ^= 0%; *P* = 0.56) by random effects, as shown in Figure 6C.

#### Readmission rate

**Figure 7:**
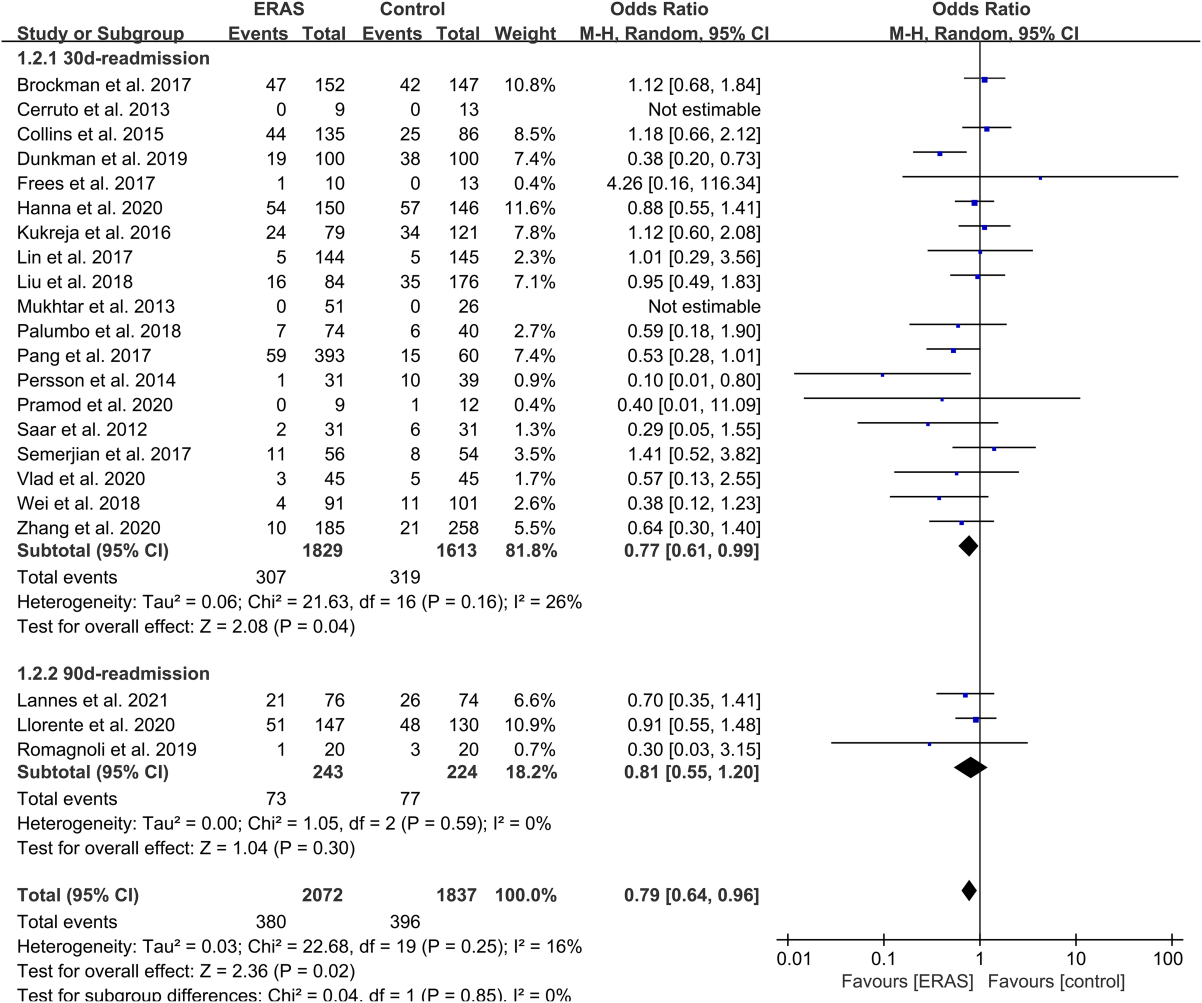
Meta-analysis and subgroup analysis of readmission rate between the ERAS and control group. ERAS, enhanced recovery after surgery; CI, confidence interval.

A total of 22 included studies reported the rate of readmission. Of these studies, 19 mentioned the 30-day readmission (15, 16, 18–21, 25–34, 36–38), and three mentioned the 90-day readmission (23, 24, 35). Therefore, we conducted a subgroup on readmission rate, which showed that the OR value of the 30-day readmission was 0.77 (95% CI: 0.61–0.99; *P* = 0.04) with low heterogeneity (*I*^2 ^= 26%; *P* = 0.16) by random effects. The OR of the 90-day readmission was 0.81 (95% CI: 0.55–1.20; *P* = 0.30) with low heterogeneity (*I*^2 ^= 0%; *P* = 0.59), and the OR of the total readmission was 0.79 (95% CI: 0.64–0.96; *P* = 0.02) with low heterogeneity (*I*^2 ^= 16%; *P* = 0.25), as shown in Figure 7. No publication bias was found using Egger's test (*P* = 0.097).

#### Mortality

**Figure 8:**
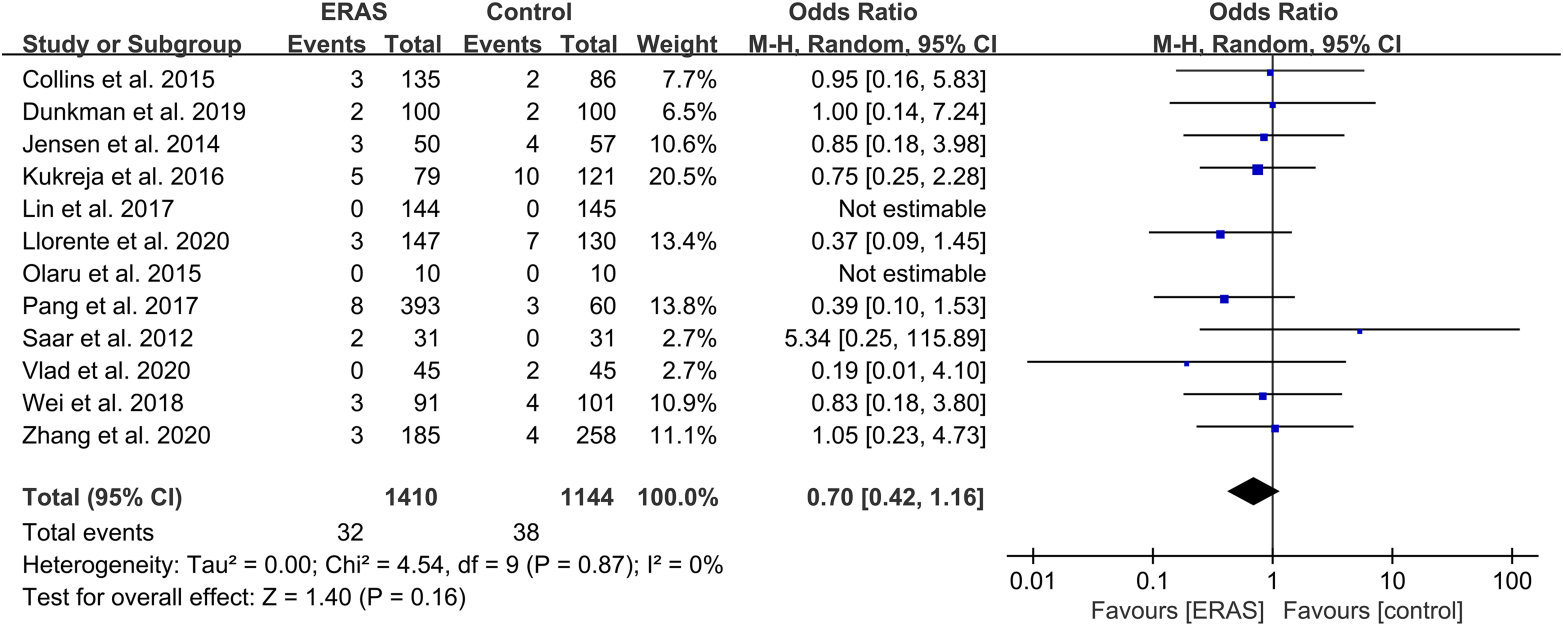
Meta-analysis of 90-day mortality between the ERAS and control group. ERAS, enhanced recovery after surgery; CI, confidence interval.

A total of 12 studies reported 90-day mortality (16, 20–23, 27, 29–33, 39), with 32 deaths (2.3%) in the ERAS group and 38 deaths (3.3%) in the control group. The pooled OR value was 0.70 (95% CI: 0.42–1.16; *P* = 0.16) with low heterogeneity (*I*^2 ^= 0%; *P* = 0.87), as shown in Figure 8. This result indicated no significance between the two groups.

#### Transfusion rate

**Figure 9:**
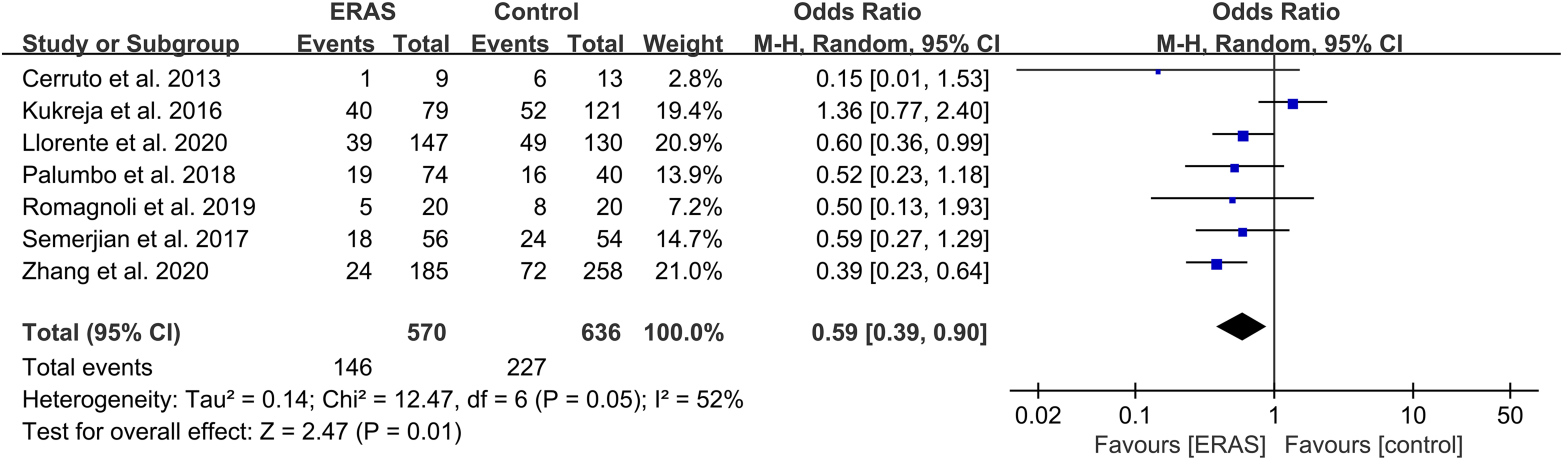
Meta-analysis of transfusion rate between the ERAS and control group. ERAS, enhanced recovery after surgery; CI, confidence interval.

A total of 12 studies reported the transfusion rate (15, 18, 19, 21, 23–25, 29, 31, 33, 35, 37), and a meta-analysis with seven studies that excluded the differences in surgery was conducted (15, 21, 23, 25, 31, 35, 37). The pooled OR was 0.59 (95% CI: 0.39–0.90; *P* = 0.01) with moderate heterogeneity (*I*^2 ^= 52%; *P* = 0.05), as shown in Figure 9. No publication bias was found using Egger's test (*P* = 0.553).

### Sensitivity analysis

We conducted the sensitivity analysis by omitting individual studies sequentially. According to the meta-analysis of each group, the aggregated OR of the remaining studies did not exceed the estimated range, as shown in Supplementary Figure S2. Furthermore, no material differences were found between the adjusted and preliminary aggregated estimates, which showed that our meta-analysis exhibited strong robustness.

## Discussion

Through our meta-analysis, we found that patients with the implementation of the ERAS program had a lower risk of readmission, overall complications, and POI. For the intraoperative situation, we found that the implementation of ERAS was beneficial in reducing the intraoperative blood transfusion rate in similar surgical procedures (21, 23), which may lead to the optimization of the intraoperative fluid volume and the use of local anesthesia. A study conducted by Linder et al. (40) indicated that the reduction of blood transfusion might reduce cancer recurrence and mortality after radical cystectomy. No significant difference in urine leakage and mortality was shown.

Direct analysis of the studies including the data on LOS showed that LOS was significantly shorter in the ERAS group, which was concordant with other studies (7, 41). Our study may show a higher level of rank relative to the transformed evidence above. This benefit has also been demonstrated in other surgical disciplines, such as thoracic (42) and colorectal surgery. It is worth mentioning that univariate and multivariate analyses were conducted to analyze the factors related to LOS in the study of Karl H. Pang et al. (33), which showed that the ERAS program was a strong influencing factor in decreasing LOS.

For the analysis of complications, a significantly lower incidence of complications was shown, which may validate the hypothesis that ERAS reduced complications. Analyses involving the data on readmission could demonstrate that the implementation of ERAS decreased the rate of readmission, which was consistent with the reduction of overall complications. POI was one of the main postoperative complications, and the first time to defecation and flatus was shorter than that of traditional regimes, which indicated that ERAS could enhance bowel function and reduce the incidence of POI.

The conclusions drawn in our study are partly consistent with those in some studies (7, 41). Our study supported their findings on LOS, POI, and time to defecation, which had inconsistent outcomes on readmission and overall complications. Our outcomes show more beneficial results for ERAS than those of the mentioned studies, but some limitations were identified due to the diversity of research types rather than with RCTs only. As far as we are concerned, RCTs may have a better level of evidence, despite their limited number and small amount of data. Hence, the inclusion of prospective and retrospective studies may increase the amount of data and reliability of the study. In our opinion, more additional RCTs should be conducted to explore the effect of ERAS on radical cystectomy and further investigate the function of the ERAS elements on complications to optimize choices in the clinic.

Since the publication of ERAS guidelines (6), 22 items cannot be fully implemented due to the limitations of each hospital. Therefore, it was necessary to identify the value of every ERAS element, to optimize ERAS for better application. For example, 22/25 studies carried out preoperative counseling and education, which proved this item could well be adopted due to the reduction of postoperative anxiety and depression, as reported in some studies (43). All studies conducted the early oral diet, and two studies (19, 38) omitted the early mobilization. Prevention of POI focused on chewing gum and oral magnesium, as well as oral metoclopramide and alvimopan, also showed benefits. Other elements also got approved in some studies, such as carbohydrate loading, which, as proven by Svanfeldt et al. (44), could shorten LOS and improve gut function due to the reduction of insulin resistance and thirst (45).

Not only does the benefit of each element need attention but also the polymorphism that ERAS brings to patients. Our study indicated that the multimodal nature of ERAS might surpass the attention to a single element in perioperative outcomes.

The possible limitations that existed in our study were the limited number of RCTs and only a blinded RCT. Other RCTs had at least an unclear bias in one domain. Therefore, the evidence level may be lower than that of those studies that relied on conclusions drawn from RCTs. The other limitation was that we did not perform a subgroup analysis of surgical and urethral diversion methods, which may introduce some bias. Finally, our study did not include an analysis of health economics and quality of life. Our study indicates that the implementation of ERAS protocols was beneficial in decreasing the overall complication and readmission compared with conventional protocols, which were inconsistent with other studies but showed the benefits of ERAS. Furthermore, the perioperative outcomes of radical cystectomy after the conducted ERAS showed better improvement in LOS, bowel function, and blood transfusion rate. These data are statistically significant in clinical value and promote the clinical application of ERAS to help patients recover smoothly after radical cystectomy.

## Conclusion

ERAS can reduce overall complications and readmission and transfusion rates and can shorten the time to flatus, defecation, and LOS after radical cystectomy.

## Data Availability

The raw data supporting the conclusions of this article will be made available by the authors, without undue reservation.
