# Supplementary material for: The effect of the enhanced recovery after surgery program on radical cystectomy: a meta-analysis and systematic review
Source: Front Surg. 2023 May 19;10:1101098. doi: 10.3389/fsurg.2023.1101098 (PMC10235530; doi:10.3389/fsurg.2023.1101098)
Supplement: Supplementary file 1 [file Datasheet1.pdf]

Table S1. Details of search strategy for all databases

Database	Search Strategy
PubMed	((((("urinary bladder neoplasms"[MeSH Terms] OR ("urinary"[All Fields] AND "bladder"[All Fields] AND "neoplasms"[All Fields]) OR "urinary bladder neoplasms"[All Fields] OR ("bladder"[All Fields] AND "neoplasm"[All Fields]) OR "bladder neoplasm"[All Fields]) OR ("bladder tumour"[All Fields])) OR ("bladder neoplasm"[All Fields])) OR ("bladder cancer"[All Fields])) OR (("cystectomy"[MeSH Terms] OR "cystectomy"[All Fields]) OR ("urinary diversion"[MeSH Terms] OR ("urinary"[All Fields] AND "diversion"[All Fields]) OR "urinary diversion"[All Fields]))) AND (((((("enhanced recovery after surgery"[MeSH Terms] OR "enhanced recovery after surgery"[All Fields]) OR (eras)) OR (accelerated rehabilitation)) OR (intensive rehabilitation)) OR (fast track)) OR (enhanced recovery))
Embase	(('enhanced recovery' OR 'fast track' OR 'intensive rehabilitation' OR 'accelerated rehabilitation' OR 'eras' OR 'enhanced recovery after surgery') AND ('bladder neoplasm' OR 'bladder tumor' OR 'urinary bladder neoplasm' OR 'bladder cancer' OR 'bladder cancer'/exp OR 'urinary diversion' OR 'urinary diversion'/exp OR 'cystectomy' OR 'cystectomy'/exp))
Scopus	(ALL (('bladder AND neoplasm' OR 'bladder AND tumor' OR 'urinary AND bladder AND neoplasm' OR 'bladder AND cancer' OR 'bladder AND cancer' OR 'urinary AND diversion' OR 'urinary AND diversion' OR 'cystectomy' OR 'cystectomy')) AND ALL ((‘enhanced AND recovery’ OR ‘fast AND track’ OR ‘intensive AND rehabilitation’ OR ‘accelerated AND rehabilitation’ OR ‘eras’ OR ‘enhanced AND recovery AND after AND surgery’)))
Cochrane library	(((Urinary Bladder Neoplasms[MeSH]) OR ((Bladder) AND (NEOPLAS* OR CANCER OR CARCINOMA*))) OR ((Cystectomy OR （urinary diversion)）) AND ((Enhanced Recovery After Surgery) OR (eras) OR (accelerated rehabilitation) OR (intensive rehabilitation) OR (fast track) OR (enhanced recovery))
