# Supplementary figures and images for: The effect of the enhanced recovery after surgery program on radical cystectomy: a meta-analysis and systematic review
Source: Front Surg. 2023 May 19;10:1101098. doi: 10.3389/fsurg.2023.1101098 (PMC10235530; doi:10.3389/fsurg.2023.1101098)

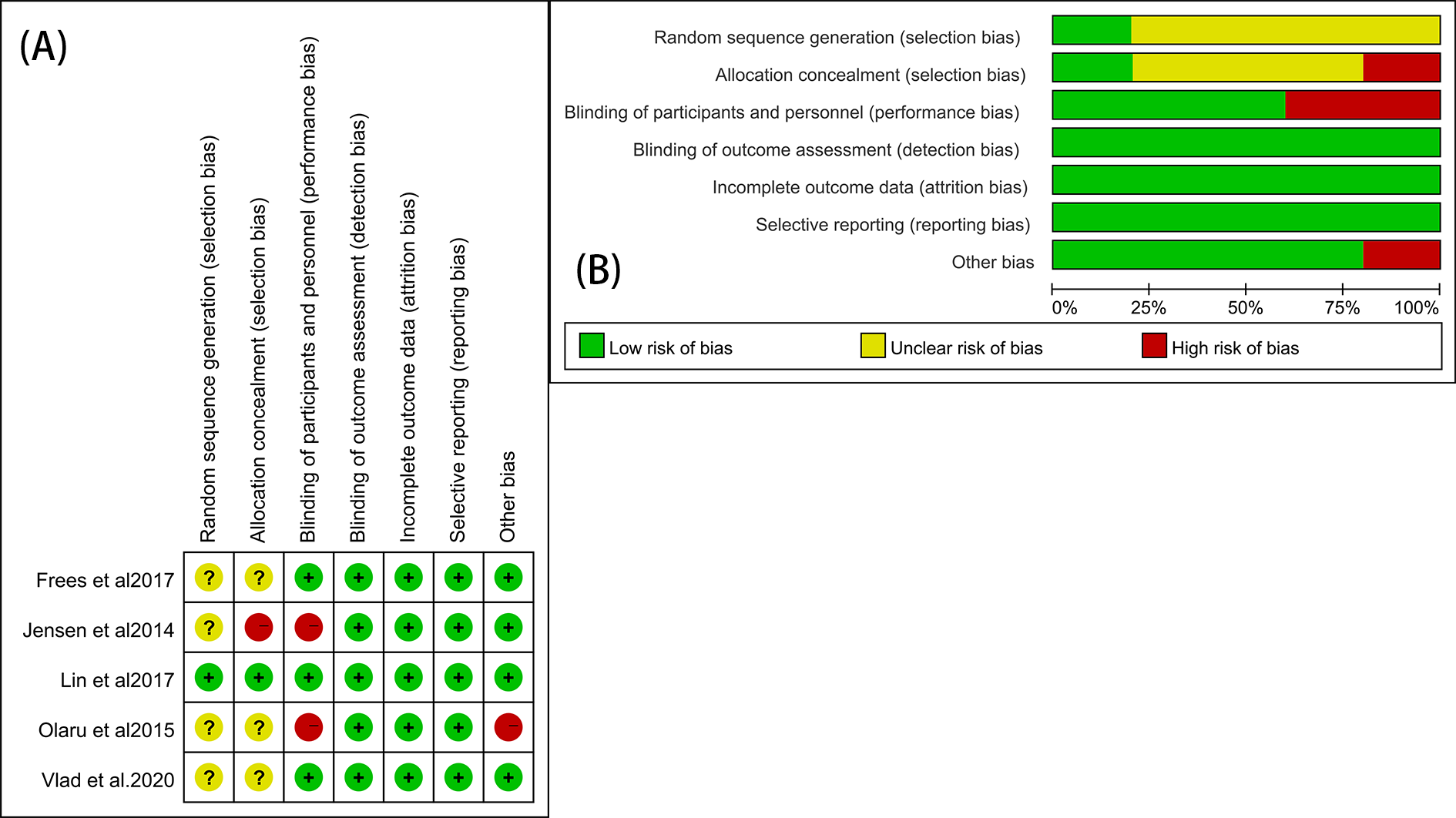

Supplement: Supplementary file 2 [file Image1.tif]

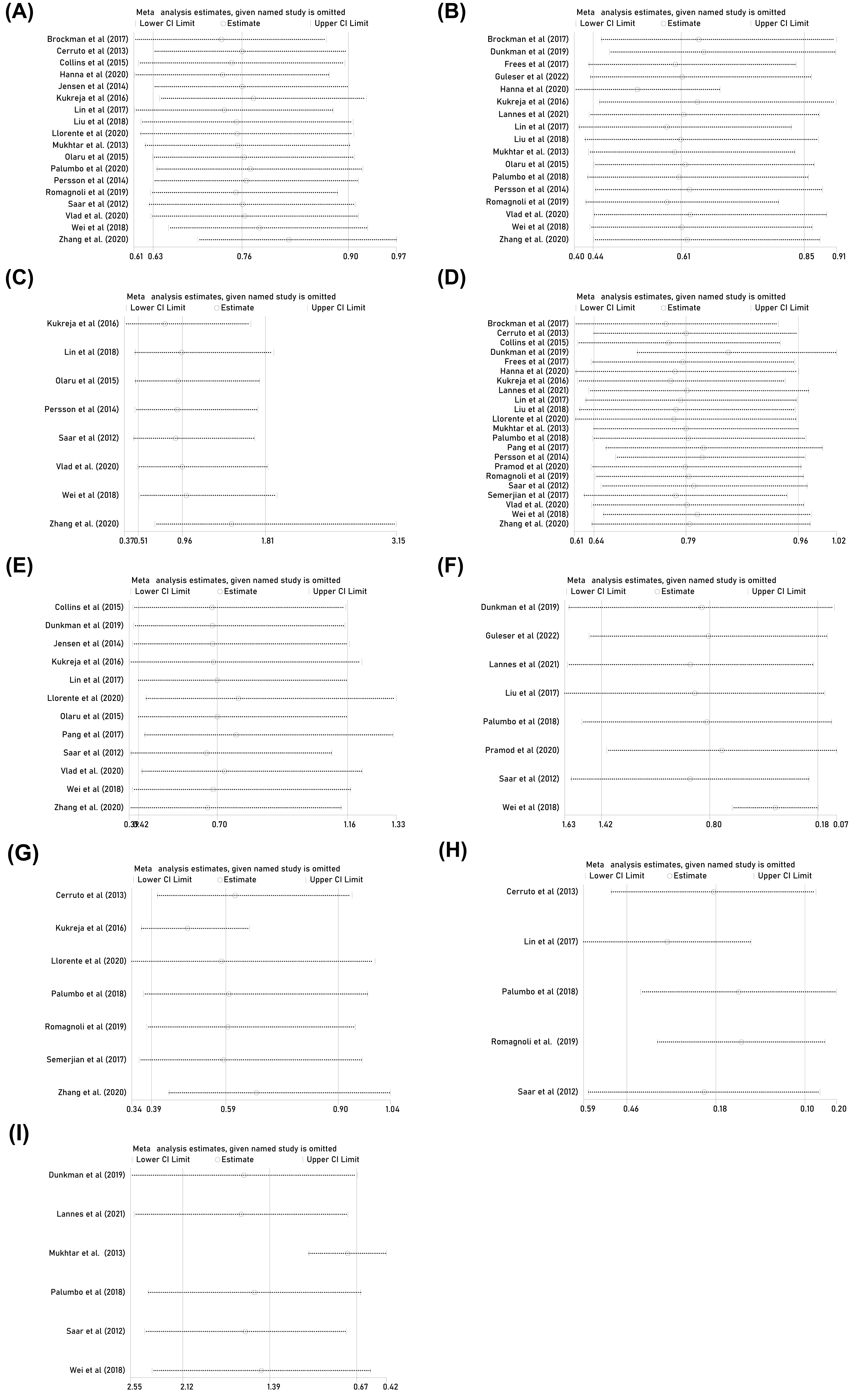

Supplement: Supplementary file 3 [file Image2.tif]
